# Determinants of Overall Satisfaction with Public Clinics in Rural China: Interpersonal Care Quality and Treatment Outcome

**DOI:** 10.3390/ijerph16050697

**Published:** 2019-02-27

**Authors:** Wenhua Wang, Elizabeth Maitland, Stephen Nicholas, Jeannie Haggerty

**Affiliations:** 1 Department of Family Medicine, McGill University, Montreal, QC H3T 1M5, Canada; wenhua.wang@mail.mcgill.ca (W.W.); jeannie.haggerty@mcgill.ca (J.H.); 2 University of Liverpool Management School, University of Liverpool, Liverpool L697ZH, UK; e.maitland@liverpool.ac.uk; 3 School of Management and School of Commerce, Tianjin Normal University, Tianjin 300074, China; 4 Guangdong Research Institute for International Strategies, Guangdong University of Foreign Studies, Guangzhou 510420, China; 5 TOP Education Institute, Sydney, NSW 2015, Australia; 6 University of Newcastle Business School, Newcastle, NSW 2308, Australia

**Keywords:** public clinics, patient satisfaction, patient centeredness, interpersonal care quality, treatment outcome, primary health care, China

## Abstract

The primary health care quality factors determining patient satisfaction will shape patient-centered health reform in China. While rural public clinics performed better than hospitals and private clinics in terms of patient perceived quality of primary care in China, there is little information about which quality care aspects drove patients’ satisfaction. Using a World Health Organization database on 1014 rural public clinic users from eight provinces in China, our multiple linear regression model estimated the association between patient perceived quality aspects, one treatment outcome, and overall primary health care satisfaction. Our results show that treatment outcome was the strongest predictor of overall satisfaction (β = 0.338 (95% CI: 0.284 to 0.392); *p* < 0.001), followed by two interpersonal care quality aspects, Dignity (being treated respectfully) (β = 0.219 (95% CI: 0.117 to 0.320); *p* < 0.001) and Communication (clear explanation by the physician) (β = 0.103 (95% CI: 0.003 to 0.203); *p* = 0.043). Prompt attention (waiting time before seeing the doctor) and Confidentiality (talking privately to the provider) were not correlated with overall satisfaction. The treatment outcome focus, and weak interpersonal primary care aspects, in overall patient satisfaction, pose barriers towards a patient-centered transformation of China’s primary care rural clinics, but support the focus of improving the clinical competency of rural primary care workers.

## 1. Introduction

China launched an ambitious national health reform plan in 2009 with the aim of providing universal coverage of essential health services that are safe, effective, accessible, and affordable for all Chinese citizens by 2020 [1]. With a start-up investment of USD 125 billion, this health reform reaffirmed the government’s central role in financing health care services, which focused on strengthening the primary care system, establishing a national essential medicines system, expanding health insurance coverage, providing public health services, and conducting public hospital reform [2,3]. Notable progress has been achieved since then. The population coverage of social health insurance schemes increased to more than 97% in 2015, and the share of the out-of-pocket expenditure in total health expenditure decreased to less than 30% by 2016 [2]. Initially piloted at primary health care centers, the zero-markup drug policy was rolled out at county hospitals in the beginning of 2012 and in city hospitals in 2015 [4]. To improve primary care service delivery capacity, the government increased its subsidies to primary health care institutions from USD 2.8 billion in 2008 to USD 20.3 billion in 2015 [5]. Recently, China’s 13th five-year plan for health reform reconfirmed the central government’s commitment to build a primary health care-based integrated health system [6].

The primary health care system in rural China is mainly organised around village clinics and township health centers, although county hospitals also provide primary care, as well as specialist medical services. Village clinics, generally located in each village, include 2–3 health staff, with only a short period of medical training, and 12% of doctors’ educational levels were below the official requirements for village doctors (three years of technical school education after nine years of primary and secondary education) [5,7]. Providing primary care and public health services, township health centers average 34.5 medical staff, and 31% of doctors’ educational levels were below the requirement for a licensed assistant doctor (junior medical college degree) [5,7,8]. Among the 37,000 township health centers, 99% are publicly owned, while 63% of 639,000 village clinics are public and 37% privately-owned. The 2013 fifth national health service survey showed that over 80% of patients visited township health centers or village clinics as their first choice for primary health care [9]. 

Research on strengthening primary health care explores innovative organizational models of service provision, focusing on patient-centered care, which calls for health services that are organized around the health needs and preferences of individuals. This requires active patient engagement in the care seeking process; coordinates and integrates health care across different levels; provides education and information; and ensures access, continuity and transition in health care [10,11,12]. Substantial research evidence shows the significant benefits of developing patient-centered health care systems, most importantly increased patient satisfaction and empowerment, but also the reduction of malpractice complaints, improvements in the adherence to recommended clinical practice and medication, less health care utilization and reduced health care costs [13,14,15]. Comparison studies of patient experience performance have been conducted in many countries to identify optimal primary care delivery models. In Africa, private healthcare facilities provide more responsive care than public providers in Ghana and South Africa, especially in timeliness and respect [16,17]. In Latin America, the overall primary care performance of Argentina’s social security subsystem performed better than either the public or private subsystems [18]. In Asia, patients receiving care from private general practitioners in Hong Kong reported better primary care experiences than those from public general outpatient clinics, especially in accessibility and interpersonal relationships [19]. In South Korea, among four types of primary care clinics staffed by family physicians, health cooperative clinics displayed the best primary care performance, while public health center clinics showed the worst performance [20]. 

In rural China, as the first call for patient-centered primary health care, village clinics and township health centers generally perform better interpersonal care than hospitals, especially in accessibility, communication, family centeredness, and community orientation, whether in developed areas, such as the Guangdong province [21] or in less developed regions, such as Tibet [22]. Using national representative data, a previous study showed that public clinics perform better than private clinics in rural China, in patient perceptions of accessibility, communication, autonomy, privacy, and respect [23]. These suggest that public clinics might be the optimal organizational model of primary care provision in rural China. 

China has also made steps in the direction of patient-focused care. For example, the family doctor contract service model was developed and expanded to build a gate-keeper role for primary care clinics based on a long-term therapeutic relationship between family doctors and patients [24]. The transformation of the multi-tiered diagnosis and treatment system aimed to promote collaboration between different healthcare providers and to improve the coordination system between hospitals and primary care clinics for integrated care [2,25]. These policy interventions will have a significant impact on the organization and operation of public clinics in rural China. Clear information of population preference would help guide the policy implementation in practice and facilitate this transformation process. However, there is little information about what matters most to public clinic users in rural China. Which care aspects drove patients’ overall satisfaction with rural public clinics?

Various approaches have been used to elicit patient preferences or priorities for health care. Identifying health care aspects that show the strongest relationship with overall satisfaction is one approach [26]. As part of the health outcome quality [27], an expression of satisfaction or dissatisfaction is also the patients’ judgement on the quality of care in all its aspects, but particularly on interpersonal and technical aspects of care [28]. While widely studied in developed countries since the 1970s and 1980s [29,30,31,32,33], patient experience and satisfaction studies have only recently been of interest in China. The few China-based studies have mostly been conducted in hospitals settings, especially in tertiary hospitals in developed areas [34,35,36,37,38,39]. Understanding the relationship between patient perceived care quality and satisfaction would point to those care aspects that matter most to patients, which could guide primary health care resource allocation and service delivery reorganization. In this study, we aimed to identify those care aspects perceived by patients, as the most important factors driving overall satisfaction at public clinics in rural China. We also make policy recommendations on how China’s primary health care delivery system can be improved, and resources more optimally allocated.

## 2. Methods

### 2.1. Data Source 

The World Health Organization (WHO) Study on global AGEing and adult health (SAGE) is a longitudinal study with nationally representative samples of persons aged 50 years and older in China, Ghana, India, Mexico, the Russian Federation and South Africa, with comparison samples of younger adults aged 18–49 years in each country [40]. Based on a multistage cluster sampling design; face-to-face interviews were conducted by trained interviewers to collect information on socio-demographics, health risk factors and chronic conditions, health service utilization and patient responsiveness using a standardized instrument. The SAGE China Wave 1 was completed in 2010, including data on 14,813 respondents. The overall response rate was over 98% [41,42,43]. 

To be included in this study, the eligible participants should meet the following three criteria: (1) living in rural China, (2) having received outpatient care over the last 12 months, (3) using a rural public clinic for the last outpatient care. In summary, 7673 participants were from rural China, of whom 3435 had received outpatient care over the last 12 months. Among the 3435 participants, 1014 of them used rural public clinics for the last outpatient care. Therefore, 1014 eligible participants were included in our final analysis. Since study participants were selected using a randomized sampling method, the data of eight provinces provided a representative sample of public clinic users in rural China.

### 2.2. Measurement

#### 2.2.1. Interpersonal Care Quality and Treatment Outcome

The main explanatory variables of overall satisfaction in this study are patient-perceived health care quality. Interpersonal, or patient-centered, care quality measures and treatment outcomes were selected based on data availability and Jackson’s satisfaction model, which suggests that satisfaction, measured outside the context of an immediately completed patient visit, needs to take into account symptom-specific improvements [44]. In response to this finding, we included both the treatment outcome and interpersonal quality variables in our regression model, besides patient socio-economic and demographic characteristics. 

From the WHO SAGE Survey, prompt attention, dignity, communication, autonomy, and confidentiality were five domains used to measure interpersonal care quality [45,46]. Outpatients were asked to rate from 1 = very bad to 5 = very good, their most recent health care visit based on: (1) Prompt attention: the amount of time you waited before being attended; (2) Dignity: your experience of being treated respectfully; (3) Communication: how clearly health care providers explained things to you; (4) Autonomy: your experience of being involved in making decisions for your treatment; and (5) Confidentiality: the way the health services ensured that you could talk privately to providers. In previous studies, the five questions from patient responsiveness surveys developed by the WHO showed adequate psychometric properties [46].

The treatment outcome was measured using one question “What was the outcome of your visit to the health care provider? With five Likert response options ranging from 1 = get much better to 5 = get much worse. For easy interpretation, we reversed the response in our analysis. 

#### 2.2.2. Overall Satisfaction

To measure overall patient satisfaction, a single-item “Overall, how satisfied were you with the care you received during your last visit?” Had five Likert response options, ranging from 1 = very satisfied to 5 = very dissatisfied. For easy interpretation, we reversed the response in our analysis. 

### 2.3. Patient Characteristics

Demographic and socioeconomic variables included age, gender, education and income quintile. Health status variables included self-rated health and the presence of chronic conditions. Self-rated health included five categories: very good, good, moderate, bad and very bad. Using eight common chronic conditions (angina, arthritis, asthma, stroke, diabetes, depression, chronic lung disease, and hypertension) listed in the survey, the presence of chronic conditions was categorized into none (1), one (2) and two or more (3) [38]. Age was dichotomized into 18–59 years versus 60+ years old. Education included four categories: less than primary school, primary school, secondary school, and high school or above. The income quintiles were based on the possession of a set of household assets and a number of dwelling characteristics with Q1 representing the poorest household category and Q5 representing the richest household category [41,42,43]. 

### 2.4. Statistical Analysis

In user-evaluation research, it is common to treat report and rating values as quasi-cardinal [47]. Consistent with the analytical approach of previous psychometric analysis of WHO’s health system responsiveness questions, the items measuring interpersonal care quality, treatment outcome and overall satisfaction in this study were treated as interval-level data [46].

Using SPSS 22.0 (IBM, Armonk, NY, USA), statistical analysis took the following three steps. First, demographic characteristics of participants visiting rural public clinics was described using percentages, and the performance of interpersonal care quality, treatment outcome and overall satisfaction was described using means. Second, Pearson correlation was used to examine the correlation between each care aspect of patient perceived quality and overall satisfaction. Third, a multiple linear regression model was used to examine the association between patient perceived quality and overall satisfaction after controlling for age, gender, education, income, self-rated health, and presence of chronic conditions. Statistical significance level is *p* = 0.05 in this study.

To ensure the robustness of our regression model, several tests were conducted to check if the assumptions of the multiple linear regression model were met in our data. First, Q-Q plot, which compares the observed quantile with the theoretical quantile of a normal distribution, was created to check the normality of the standardized residual. The Q-Q plot followed the normality line well, although not perfectly. There is a little bit of deviation, but there are no drastic deviations. The normality was further confirmed by One-Sample Kolmogorov-Smirnov Test (mean = 0.000 and standard deviation = 0.991; *p* < 0.0001). Second, a scatter plot between the standardized predicted value and the standardized residual was created to check the homoscedasticity, which suggested that there was a slight violation of Homoscedasticity. The heteroscedasticity was further confirmed by the Breusch-Pagan/Cook-Weisberg test (Chi-square = 19.40, *p* < 0.001). To ensure that the slight violation of homoscedasticity would not influence the final results, a more robust method was used to calculate the standard error of the regression coefficient of each independent variable, which showed very similar results with the initial analysis. Therefore, we assumed that homoscedasticity assumption was satisfied if we run the fully specified predictive model. Third, multicollinearity was checked using the value of the Variance Inflation Factor (VIF), which indicates the degree that the variances in the regression estimates are increased due to multicollinearity. VIF values higher than 10 indicate that multicollinearity is a problem. For our regression model, the VIF values of all independent variables are lower than 10, ranging from 1.100 to 4.345. Therefore, we concluded that there was no multicollinearity in our data. In summary, the assumptions of linear regression model were satisfied in our data.

Considering most of the variables (overall satisfaction, interpersonal care quality, treatment outcome) are ordinal variables using a five-point Likert scale, we conducted sensitivity analysis using a logistic regression model. First, an ordinal logistic regression model was built. However, the test of parallel lines (Chi-square = 112.13, *p* < 0.001) showed that the proportional odds assumption was violated in our data. Then, the five-point Likert scale of the dependent variable (overall satisfaction) was dichotomized into “satisfied” (including “very satisfied” and “satisfied”) and “dissatisfied” (including “moderate”, “dissatisfied” and “very dissatisfied”). A binary logistic regression model was created, and showed the same results with the multiple linear regression model.

### 2.5. Ethical Approval

SAGE has been approved by the World Health Organization’s Ethical Review Committee. In addition, each partner organization implementing SAGE obtained ethical clearance through their respective review bodies. Written informed consent was obtained from all study participants.

## 3. Results

Demographic characteristics, self-rated health, and chronic conditions of the total 14,813 respondents are reported in Table 1 along with the characteristics of the 1014 rural public clinic users included in our analysis.

Table 2 presents the means of six care aspects of patient perceived quality and overall satisfaction on a scale of 1 very bad to 5 very good. From the patients’ perspective, public clinics in rural China perform the best in treatment outcomes (4.26 ± 0.02), followed by dignity (4.17 ± 0.02) and prompt attention (4.15 ± 0.02), while communication (4.07 ± 0.02), autonomy (4.05 ± 0.02), and confidentiality (4.02 ± 0.02) lagged behind. The score of overall satisfaction was 4.03 ± 0.02.

In Table 3 the Pearson correlation coefficient between each care aspect and the overall satisfaction shows that the coefficient was the highest in treatment outcome (0.398), followed by dignity (0.261), communication (0.226), prompt attention (0.187), confidentiality (0.167), and autonomy (0.137).

Multiple linear regression modeling confirmed that treatment outcome was the strongest predictor of overall satisfaction (β = 0.338 (95% CI: 0.284 to 0.392); *p* < 0.001). Among the five interpersonal care aspects, Dignity (being treated respectfully) (β = 0.219 (95% CI: 0.117 to 0.320); *p* < 0.001) and Communication (clear explanation by the physician) (β=0.103 (95% CI: 0.003 to 0.203); *p* = 0.043) were also major determinants of overall satisfaction. However, there was no significant association between Prompt attention (waiting time) and Confidentiality (talk privately) with overall satisfaction. Table 4 also shows that older patients and patients with higher self-rated health status had higher overall satisfaction with their health care.

## 4. Discussion

Using a national representative sample of public clinic users in rural China, we found that treatment outcome was a stronger predictor of overall satisfaction with primary care than interpersonal care aspects. Among the interpersonal care aspects, Dignity (being treated respectfully) and Communication (clear explanation by the physician) were also positively associated with overall satisfaction, but Prompt attention (waiting time before seeing the doctor) and Confidentiality (talking privately to the provider) were not correlated with overall satisfaction.

Although there was increasing attention to delivering good interpersonal care in China, like reducing waiting times, showing respect for patients, and protecting patients’ privacy, patients in rural public clinics cared most about symptom improvement or treatment outcomes. This is consistent with research findings from other developing countries, like South Africa and Liberia, where patients were prepared to tolerate negative interpersonal care aspects, such as a long waiting time, poor staff attitudes and disrespectfulness, if they received good clinical quality care that they expected, like receiving the medicine they needed and a thorough examination [48,49]. This suggests that strengthening the clinical competency of primary care health professionals and improving clinical quality should be the first priority in countries with less qualified health professionals in primary care organizations, like China.

Highly qualified primary care physicians are the cornerstone of high functioning primary care systems. However, most of the primary care health professionals in rural China received at most three years junior medical college or technical school education without residency training. In rural township health centers, only 5.9% of healthcare professionals held a bachelor’s degree or above [8]. Poor training meant that community residents did not have confidence in the clinical competency of their primary care providers. This explains why many people bypassed the rural clinics and visit hospitals directly, even for minor illness [5]. Healthcare provider education and qualifications are also strongly correlated with quality of care [50,51]. Using a standardized patient, a study from Sichuan, Shaanxi, and Anhui provinces showed that only three of 33 village doctors and 15 of 104 township doctors correctly treated diarrhoea cases. In treating angina, 33% of village doctors and 41% of township doctors failed to correctly refer patients [50]. In addition, the motivation of doctors to provide quality health care declined with the 2009 national health reforms. Ending the 15% mark up on western medication sales, first introduced into primary care clinics before being rolled out across all tiers of China’s health system, meant doctors could no longer supplement their low incomes through over-prescribing drugs and over-servicing patients, reducing the financial incentive to provide quality health care. Our findings endorse China’s ambitious program to upgrade and train over 500,000 general practitioner assistants and general practitioners over the next decade to improve the doctor quality and enhance public trust in primary care.

Following treatment outcomes, two interpersonal care aspects, Dignity (being treated respectfully) and Communication (clear explanation by the physician), were also priorities for patients in rural China. Our quantitative results are consistent with a recent qualitative study exploring patient priorities of health care in China, which showed that good communication skills of health professionals and respect for their views were appreciated by many patients [52]. A seminal systematic review on patient priorities for good primary care [53] covering papers published from 1966 to 1995 found that humaneness (respect and personal interest for the patient as an individual) was the top priority for patients in developed countries, followed by clinical competence and technical quality. Furthermore, studies in North America and Europe showed that not only respect and physician-patient communication were important for patients, but also involvement in treatment decisions and confidentiality of information [53,54]. In contrast, our study found Prompt attention (waiting time before seeing the doctor) and Confidentiality (talking privately to the provider) were not correlated with overall satisfaction, and Autonomy (involved in making decisions) was negatively correlated with overall satisfaction. The relationship between patient involvement and satisfaction is complicated, and studies of this relationship have yielded inconsistent results [55]. Increased patient involvement may lead to satisfaction, but it may also be the result of unsatisfactory or incomplete care [55]. We also know some patients prefer an active role in the decision-making process, while others prefer that physicians take the responsibility for all judgement. However, little is known about the other characteristics of such patients [55]. The existing literature suggests that patient preferences or priorities are associated with various socio-cultural and demographic factors, like age, occupation, income, and ethnicity [56]. Further comparative analysis of patient priorities in different countries from cultural perspective might help to better understand these differences.

Previous studies show that poorer physical health status, disability, low quality of life, and psychological distress are associated with lower levels of satisfaction, and older respondents were significantly more satisfied [27,44]. Our study confirmed for rural China the conventional wisdom that older patients and patients with higher self-rated health status had higher overall satisfaction with primary health care. In addition, our model explained 23.8% of the variances of satisfaction, which is better than many developed country studies, where predictors of satisfaction only explained a small portion (nearly always less than 20%) of satisfaction’s variance [44]. However, future research should explore other determinants of overall satisfaction in China, which account for the other variance of satisfaction.

There are several limitations. First, only rural public clinics users were included in this study. For patients using public hospitals or private clinics, they may never have used public clinics, or they may have stopped using public clinics due to their previous experiences. A further study should include these private clinic and hospital user groups and investigate why they do not use public clinics. Second, only one item was used to measure prompt attention, communication, autonomy, dignity, and confidentiality, which may not capture the full performance information of each care aspect. Since there are very limited tools to measure patient-centered care in China, further studies should develop appropriate measurements based on the Chinese populations’ preferences and valuation of health care. Third, separate analysis for specific conditions could not be conducted because of data limitations. Patients with different health problems may have different priorities during their clinical encounter, with different satisfaction outcomes. Further study of specific patient groups in rural public clinics could provide more specific information for service delivery transformation. Fourth, this study was a secondary data analysis based on a cross-sectional survey: the WHO SAGE China Wave 1. The results from this study cannot draw conclusion about the causality between interpersonal care quality, treatment outcome and overall satisfaction. The WHO SAGE is a longitudinal study, the implementation of the full SAGE Wave 2 follow-up was completed in China by the end of 2015. After the data of China Wave 2 is publicly released, further analysis may be conducted to explore the causality between patient experience and overall patient satisfaction.

## 5. Conclusions

This study provides empirical evidence that rural public clinic users in China prioritize symptom resolution before interpersonal care quality aspects, such as respectfulness and physician-patient communication. Key patient-centered attributes of primary care utilization, waiting time and privacy, were less important in improving patient satisfaction. These findings suggest that the reform of the Chinese primary care towards a patient-centered system faces significant barriers from treatment outcome focused patients. Patient-centered health care systems are both supply and demand-side driven, and while the 2009 reforms allowed changes towards a patient-centered care model, there was little interest from patients for rapid changes towards a more patient-centered system.

There are several policy implications from this study. First, clinical competency of rural primary care workers should be the first priority, with even common diseases, like diarrhoea, incorrectly diagnosed and treated. Besides medical education reform for students in family medicine or general practice in medical schools, regular training for some specific common diseases should also be provided for rural primary care workers through a continuing medical education program and building a learning practice environment. Second, a patient-centered system is likely to be supply driven in China, depending on the improvement of interpersonal skills of rural primary care workers, which will lead to higher user satisfaction. Since the content of interpersonal care quality varies across different cultures, relevant training programs should be tailored to specific interpersonal care aspects, especially those that are important among the Chinese population. For example, which physician behaviors are linked to Chinese patients’ feeling of getting respect? What is a clear physician’s explanation from a Chinese patient’s perspective? Third, some quality metrics, including clinical quality and patient experience, should be explored and developed to monitor and evaluate the performance of the family doctor contract service model. Such quality information could guide both medical education reform and service delivery transformation. Fourth, customised health education programs might be provided to community residents, especially in less developed areas. These programs could help community residents understand the current primary care system and relevant health policies, gain familiarity with the service profiles and service teams in primary care clinics, and manage their health care seeking behavior more rationally. These programs could be provided through blended innovative education approaches, including in-person classes in the clinics, or by social media, like TV, Radio, WeChat, and Newspapers. Patient demand-side driven calls for improved interpersonal primary health care will be crucial for the patient-centered transformation of China’s primary care clinics.

## Figures and Tables

**Table 1:** Characteristics of total respondents and rural public clinics users in China n (%) ^a^.

Characteristics	Total Respondents (*n* = 14,813)	Rural Respondents (*n* = 7673)	Rural Public Clinics Users (*n* = 1014)
Gender			
Male	6887 (46.5)	3747 (49.0)	470 (46.4)
Female	7924 (53.5)	3903 (51.0)	544 (53.6)
Age			
18–59	7337 (49.5)	4050 (52.9)	506 (49.9)
60+	7474 (50.5)	3600 (47.1)	508 (50.1)
Education			
Less than primary school	2492 (21.9)	1857 (36.4)	327 (32.2)
Primary school	2862 (25.1)	1689 (33.1)	473 (46.6)
Secondary school	3192 (28.0)	1182 (23.2)	157 (15.5)
High school or above	2856 (25.1)	369 (7.3)	57 (5.6)
Income quintile			
Poorest	2809 (19.1)	1934 (25.4)	227 (22.5)
Q2	2917 (19.8)	1955 (25.7)	215 (21.3)
Q3	2939 (19.9)	1485 (19.5)	225 (22.3)
Q4	3045 (20.7)	1362 (17.9)	231 (22.9)
Richest	3031 (20.6)	869 (11.4)	112 (11.1)
Self-rated health			
Very bad	278 (1.9)	182 (1.9)	36 (3.6)
Bad	2503 (17.2)	1531 (33.0)	245 (24.2)
Moderate	6420 (44.0)	2942 (39.6)	407 (40.2)
Good	4738 (32.5)	2454 (33.0)	289 (28.5)
Very good	647 (4.4)	325 (4.4)	36 (3.6)
Chronic conditions			
0	7563 (53.0)	4389 (60.5)	537 (53.0)
1	4069 (28.5)	1968 (27.1)	331 (32.6)
2 or above	2637 (18.5)	900 (12.4)	146 (14.4)

^a^ The percentages were calculated after excluding missing values of each variable.

**Table 2:** Patient perceived quality and overall satisfaction in rural public clinics (*n* = 1014).

Healthcare Aspects	Mean ± SE
Patient perceived quality	
Prompt attention	4.15 ± 0.02
Dignity	4.17 ± 0.02
Communication	4.07 ± 0.02
Autonomy	4.05 ± 0.02
Confidentiality	4.02 ± 0.02
Treatment outcome	4.26 ± 0.02
Overall satisfaction	4.03 ± 0.02

Note: Rated on a scale of 1 to 5. The higher the score, the better the patient perceived quality. SE = Standard Error.

**Table 3:** Pearson correlation between each care aspect and overall satisfaction.

Healthcare Aspects	Overall Satisfaction
Prompt attention	0.187
Dignity	0.261
Communication	0.226
Autonomy	0.137
Confidentiality	0.167
Treatment outcome	0.398

Note: All correlation coefficients are significant at *p* < 0.01.

**Table 4:** Result of multiple linear model showing the average change in overall satisfaction associated with each care aspect rating.

Characteristics	β	95% Confidence Interval	*p* Value
Prompt attention	−0.068	−0.164, 0.028	0.167
Dignity	0.219	0.117, 0.320 ***	<0.001
Communication	0.103	0.003, 0.203 **	0.043
Autonomy	−0.163	−0.253, −0.073 ***	<0.001
Confidentiality	0.055	−0.035, 0.145	0.232
Treatment outcome	0.338	0.284, 0.392 ***	<0.001
Sex (ref. = male)	−0.018	−0.079, 0.043	0.571
Age	0.004	0.001, 0.007 **	0.006
Education (ref. = Illiterate)	-		
Primary school or less	−0.080	−0.152, −0.009 *	0.028
Secondary school	0.052	−0.053, 0.157	0.332
High school or above	−0.023	−0.166, 0.121	0.758
Income (ref. = poorest)	-		
Q2	0.032	−0.056, 0.120	0.475
Q3	0.081	−0.007, 0.169	0.072
Q4	0.007	−0.085, 0.099	0.882
Richest	0.052	−0.062, 0.166	0.375
Self-rated health	0.061	0.025, 0.098 **	<0.001
Chronic conditions (ref. = none)	-		
1	0.015	−0.050, 0.081	0.645
2	0.053	−0.038, 0.145	0.254
R^2^	23.8%		

Note: *** *p* < 0.001; ** *p* < 0.01; * *p* < 0.05. “ref.” means “reference category.”.
